# Crosstalk between microwave ablation and ferroptosis: The next hot topic?

**DOI:** 10.3389/fonc.2023.1099731

**Published:** 2023-01-13

**Authors:** Lu Yu, Min Cheng, Jie Liu, Xin Ye, Zhigang Wei, Jiamei Xu, Qi Xie, Jing Liang

**Affiliations:** ^1^ Department of Oncology, Shandong Key Laboratory of Rheumatic Disease and Translational Medicine, Shandong Lung Cancer Institute, The First Affiliated Hospital of Shandong First Medical University & Shandong Provincial Qianfoshan Hospital, Jinan, China; ^2^ School of Clinical Medicine, Weifang Medical University, Weifang, China

**Keywords:** microwave ablation, ferroptosis, heat shock protein, hypoxia-inducible factor, nuclear factor erythroid 2-related factor 2, p53

## Abstract

Microwave ablation has been one form of thermal ablation in treatments for many tumors, which can locally control unresectable tumors. Ferroptosis is iron-dependent cell death caused by the cumulative reactive oxygen species and lipid peroxidation products. Recently, increasing evidence has shown that ferroptosis might play a vital role in MWA-induced tumor suppression. In this article, we briefly illustrate the concept of ferroptosis, the related signal pathways and inducers, the basic principle of microwave ablation in killing tumors, and the key molecules released after microwave ablation. Then, we describe the cross-talking molecules between microwave ablation and ferroptosis, and discussed the potential mechanism of microwave ablation-induced ferroptosis. This review explores the therapeutic target of ferroptosis in enhancing the systemic antitumor effect after microwave ablation, providing theoretical support in combinational microwave ablation with pro-ferroptosis therapy.

## Introduction

Cell death is necessary to maintain normal tissue function and morphology (1). It is of great significance in inhibiting the excessive proliferation of tumor cells. Caspase-dependent apoptosis, that is, programmed cell death, has been considered the only form of regulated cell death (RCD) for a long time (2). However, because most tumors have congenital resistance to apoptosis, the new form of cell death instead of inducing apoptosis has gradually become the new cancer treatment strategy (3). With the deepening of cell research, we have a new understanding of the cell death process and found several new regulatory pathways and unique cell death patterns, such as pyroptosis and necroptosis. Pyroptosis is influenced by the engagement of pore-forming proteins, gasdermin D (GSDMD) (4), while necroptosis is influenced by mixed lineage kinase domain-like protein (MLKL) (5). However, ferroptosis, which was coined in 2012 (6), is a form of cell death relying on iron regulation driven by excessive lipid peroxidation (7). Among these RCDs, ferroptosis gradually received a great deal of attention because it involves in growth, development, aging, immunity, and other physiological and pathological conditions (8). Ferroptosis is mainly caused by iron-dependent lipid peroxidation. Different pathways directly or indirectly regulate glutathione peroxidase, leading to the accumulation of lipid peroxidation, and ultimately leading to cell death (7). Ferroptosis is involved in a variety of carcinogenic pathways (9). Resistance to traditional antitumor therapy in some highly aggressive tumors, such as clear cell carcinoma, is associated with ferroptosis (10). Therefore, a comprehensive understanding of the regulatory mechanism of ferroptosis is of great significance and has broad prospects in tumor treatment.

Image-guided thermal tumor ablation is a precise and minimally invasive treatment technology guided by computed tomography (CT) or ultrasound. It uses the thermal effect to directly induce irreversible damage or necrosis of tumor cells (11). As an alternative method of surgical treatment, microwave ablation (MWA) has attracted more attention in the local treatment of unresectable tumors (12). It has a broad application prospect in the treatment of liver malignancies, lung malignancies, as well as bone metastases and renal tumors (13, 14).

The changes in tumor cell death and systemic immune microenvironment induced by MWA are not fully clarified, especially whether MWA affects the ferroptosis of tumor cells needs further exploration. In this article, we briefly illustrate the concept of ferroptosis, the related signal pathways and inducers, the basic principle of microwave ablation in killing tumors, and the key molecules released after microwave ablation. Then, we describe the cross-talking molecules between microwave ablation and ferroptosis, and discussed the potential mechanism of microwave ablation-induced ferroptosis. Although there is no clear direct evidence that MWA can induce ferroptosis, it is true that some key signal molecules closely related to ferroptosis are up-regulated after MWA. Therefore, whether MWA can induce ferroptosis and whether MWA combined with ferroptosis inducers is a feasible new cancer treatment strategy deserve further discussion. This review explores the therapeutic target of ferroptosis in enhancing the systemic antitumor effect after microwave ablation, providing theoretical support in combinational microwave ablation with pro-ferroptosis therapy.

## Ferroptosis

### The ferroptosis mechanisms

**Figure 1:**
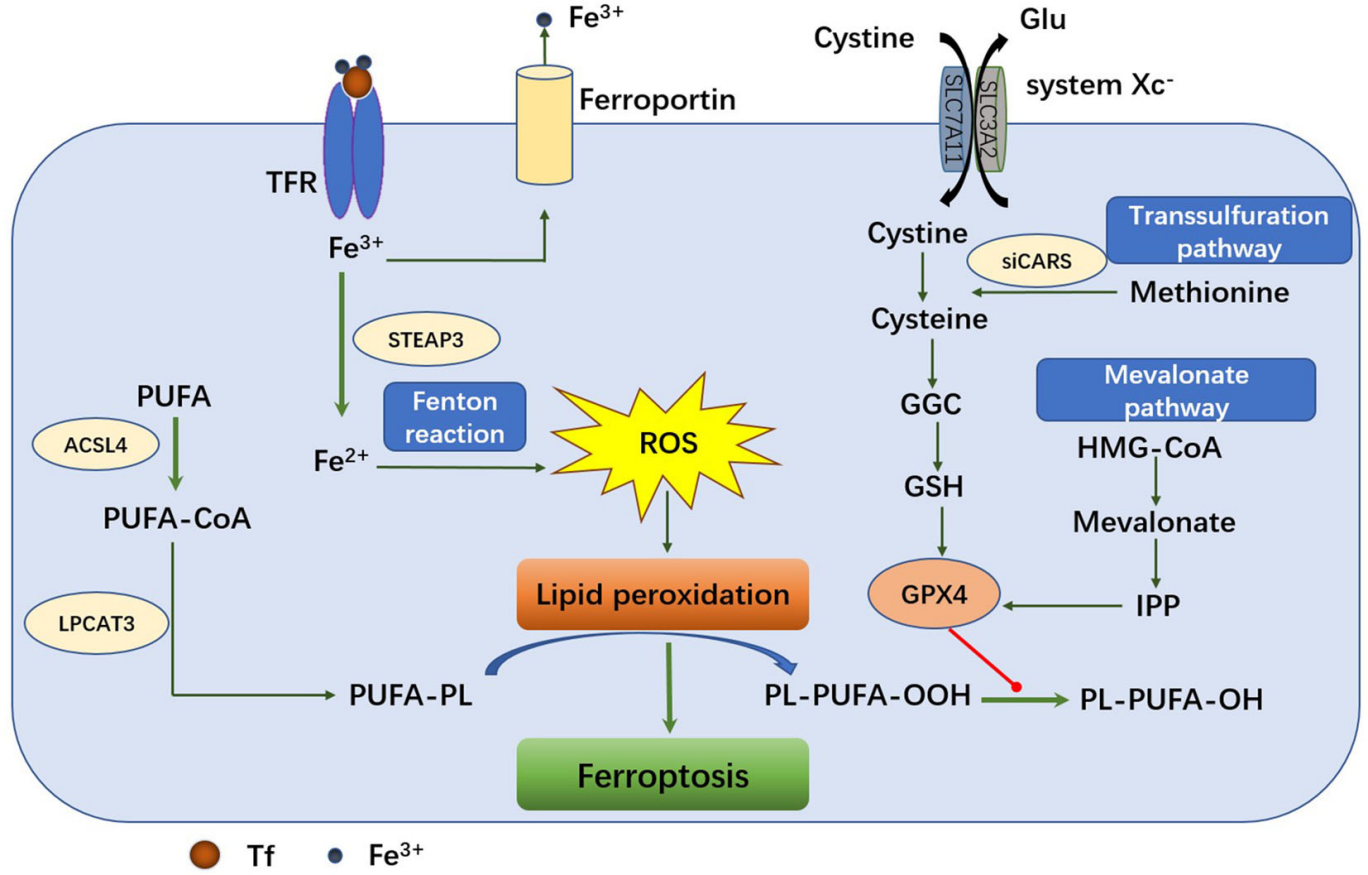
The mechanism of ferroptosis pathway. The main metabolic processes of ferroptosis can be roughly divided into three categories: iron metabolism, GSH/GPX4 pathway and lipid peroxidation. Transferrin transports the iron into cells by TFR1‐mediated endocytosis. Fe^2+^ promotes lipid peroxides accumulation through Fenton reaction and lipid oxidation. The system XC-retro delivers cystine to glutamate in a 1:1 ratio. Once inside the cell, cystine is oxidized to cysteine, which is used to synthesize glutathione (GSH) under the catalysis of glutamate-cysteine ligase (GCL) and glutathione synthase (GSS). GPX4 can reduce toxic lipid peroxides (PL-OOH) to nontoxic lipid alcohols (PL-OH) using glutathione as a reduction cofactor. Cells can also acquire cysteine by reversing the transsulfuration pathway. Another GSH/GPX4-related pathway is the mevalonate (MVA) pathway. 3-hydroxy-3-methylglutaryl-coenzyme A (HMG-CoA) can be converted to MVA in MVA pathway. Long-chain fatty acid-CoA ligase 4 (ACSL4) and lysophospholipid acyltransferase 5 (LPCAT3) promote the binding of polyunsaturated fatty acids (PUFAs) to phospholipids to form polyunsaturated fatty acid-containing phospholipids (PUFA-PLS) and then be oxidized to lipid peroxides by ROS.

The basic mechanism of ferroptosis is to regulate the balance of oxidative damage and antioxidant defense (15) (**Figure 1**). The two basic signals that cause oxidant damage are iron accumulation and lipid peroxidation (16). In this section, we have summarized the mechanism of ferroptosis and ferroptosis-inducers.

#### Iron accumulation

In general, iron balance is achieved through iron transport systems (17). Epidemiological studies have shown that excess iron can increase the incidence and risk of cancer, and experimental studies also have shown that iron is closely associated with tumorigenesis, tumor growth, invasion, and metastasis (18). The relationship between iron and tumor growth and progression is based on the role of iron in metabolism, enrichment, and metastasis (19). Iron is transported from the outside to the inside by the transferrin (TF) and transferrin receptor (TFR) (20). Imported iron is transported and stored in the shape of iron-protein complexes. The transfer of intracellular iron from cell to cell is mediated by ferroportin (FPN), which is the only iron export protein to control iron outflow (19). To accumulate iron in cells, it can increase the intake of iron or decreases iron output, promoting oxidative damage and ferroptosis in cancer cells. Iron response element-binding protein 2 (IREB2) encodes a master regulator of the metabolism of iron (6). IREB2 silencing markedly mitigated erastin-induced ferroptosis. In addition, the RAS-RAF-MEK pathway plays a final role in the sensitivity of ferroptosis in some cancer cell lines (21). One explanation is that oncogenic RAS can increase the amount of intracellular iron by decreasing ferritin and increasing TFR. Increased iron accumulation by decreasing the storage, increasing the absorption of iron, and decreasing iron outflow can promote ferroptosis through a comprehensive signaling pathway (22). There are at least two mechanisms for excess iron to promote subsequent lipid peroxidation, one is to produce reactive oxygen through an iron-dependent Fenton reaction, and the other is to activate iron containing enzymes (7).

#### Lipid peroxidation

Reactive oxygen species (ROS) including peroxide (H_2_O_2_, ROOH), superoxide (O_2_−·), singlet oxygen, and free radicals (HO·, HO_2_·, R·, RO·, NO·, NO_2_·), are molecules containing partially reduced oxygen. They can induce cell death in several ways by damaging DNA/RNA, lipids, and proteins (23). There are many sources of reactive oxygen species involved in ferroptosis, and the accumulation of oxidation products is considered the marker of ferroptosis (24). Unsaturated fatty acids and monounsaturated fatty acids are less affected by lipid peroxidation than polyunsaturated fatty acids (PUFA), so providing PUFA to cells is able to increase the sensibility of cells to ferroptosis. Free polyunsaturated fatty acids can be esterified by stimulation of acyl-CoA synthetase long-chain family member 4 (ACSL4) and pass into membrane phospholipids with lysophosphatidylcholine acyltransferase 3 (LPCAT3) (25). The up-regulation of ACSL4 is also the sign of ferroptosis (26).

#### Antioxidant defense

Macromolecular nutrients such as fat, sugar and proteins must pass the membrane of cells need the help of carriers to diffuse into the cell. System Xc− is such a transporter, which is a disulfide-linked heterodimer, composed of the regulatory subunit solute carrier family 3 member 2 (SLC3A2) and the catalytic subunit solute carrier family 7 member 11 (SLC7A11) (27). Glutamate and cystine exchanges on the plasma membrane is achieved through the system Xc−. When cystine enters the cell, it is immediately reduced to cysteine (28). Selenoenzyme glutathione peroxidase 4 (GPX4), as a central downstream regulator of ferroptosis, reduces toxic phospholipid hydroperoxide to non-toxic phospholipid (PE−AA−OH, PE−AdA−OH) by using two glutathione (GSH) molecules as electron donors, making oxidized GSH (GSSG) to combat lipid peroxidation (29). In addition to the introduction of cysteine through the system Xc−, cells acquire cysteine by reversing the transsulfuration pathway. This pathway converts methionine to S-adenosyl homocysteine and homocysteine to produce cysteine (30). The other GSH/GPX4-related pathway is the mevalonate (MVA) pathway. 3-hydroxy-3-methylglutaryl-coenzyme A (HMG-CoA) can be converted to MVA in MVA pathway. Next, MVA can be further converted to isopentenyl pyrophosphate (IPP) and CoQ10, which directly or indirectly promote GSH/GPX4 (31).

### Ferroptosis inducers

Several ferroptosis inducers (FINs) have been demonstrated to inhibit the activity of SLC7A11 and consume GSH, or inhibit the activity of GPX4 (32), or indirectly consume Coenzyme Q (CoQ) and GPX4 by activating squalene synthase (SQS) (33). In addition, developed nanomaterials have been used in local tumor for inducing ferroptosis (2). These FINs are regarded as a new potential approach to cancer treatment. The action mechanism and application of these FINs are shown in **Table 1**.

**Table 1:** Summary of ferroptosis inducers.

Agent	Target	Tumor type or cell line	Ref
Sulfasalazine (SAS)	Inhibiting system Xc-	Breast cancer, Glioblastoma, small-cell lung cancer (SCLC)	(7, 34)
Sorafenib	Inhibiting system Xc-	Renal carcinoma, Hepatocellular carcinoma, Thyroid cancer	(35)
Piperazine erastin (PE)	Inhibiting system Xc-	–	(2)
Erastin	Inhibiting system Xc-	–	(36)
Imidazole ketone erastin (IKE)	Inhibiting system Xc-	Diffuse large B cell lymphoma (DLBCL)	(37)
(1S, 3R)-RSL3	Inhibiting GPX4	Renal carcinoma	(36)
FIN56	Inhibiting GPX4	–	(38)
Glutamate	Inhibiting GPX4	DLBCL, Renal carcinoma	(33)
Altretamine	Inhibitor of GPX4	–	(39)
FINO2	GPX4 inactivation and iron oxidation	BJ-eLR cancer cells	(40)
Artemisinins	Regulating iron homeostasis	Pancreatic ductal adenocarcinoma cells (PDAC)	(41)
Dihydroartemisinin (DHA)	Autophagic degradation of ferritin	Head and neck squamous cell carcinoma, Acute myeloid leukemia	(42)
αMSH-PEG-C′	Increase intracellular iron level	Melanoma	(43)
p53 plasmid-encap-sulated metal–organic network (MON-p53)	SLC7A11 inhibition	4T1 breast cancer	(44)
Statins	Inhibiting the biosynthesis of selenoproteins and CoQ10	–	(45)
Siramesine	Overexpression FPN	Breast cancer	(46)
Lapatinib	Overexpression FPN	Breast cancer	(46)
Cisplatin	Inhibiting GSH	Pancreatic carcinoma, Oophoroma, Urothelial cancer	(47)
Fluvastatin	Inhibiting HMGCR	Breast cancer	(7)

## Microwave ablation

### The mechanism of microwave ablation

MWA has been used as a classical thermal ablation for decades (48). Guided by visual imaging, a microwave antenna can be inserted into the tumor center with precision (49). The microwave generator emits electromagnetic waves through an uninsulated tip. MWA uses the maximized agitation to flip the water molecules at 2 to 5 billion times per second in tumor tissue, depending on the energy frequency of the microwave (50). Electromagnetic microwaves create friction and heat that induces cell death through coagulation necrosis (51). Compared with other existing thermal ablation techniques, the main advantages of microwave ablation lie in continuously higher intratumor temperature, larger tumor volume, faster ablation time, and better convective distribution (52). When the probe is inserted into the tumor tissue, ablation can be divided into three areas depending on the temperature. Coagulative necrosis occurred in the central region at temperatures of ≥ 50°C (53). Due to the high temperature in this area, cell membrane damage, protein denaturation, enzyme inactivation, DNA polymerase function damage, and mitochondrial dysfunction are caused. In the periphery of the central region, also known as the transition zone, sublethal and reversible heat-induced damage occurs at temperatures between 41° C and 45° C. Cells in the subregion may be vulnerable to further damage due to metabolic dysfunction or cessation (54). Peripheral areas lead to increased oxygenation through increased blood flow, which may increase ROS and free radicals. The damaged local tissues exposed hyaluronic acid and endothelial damage markers, stimulated the expression of chemokines and vascular adhesion molecules, and attracted immune cells (55). This area contains the infiltration of a large number of inflammatory cells, including CD4^+^ and CD8^+^ T cells, natural killer cells (NKs), dendritic cells (DCs), macrophages, and neutrophils (52, 56). MWA could induce the release of tumor antigens and a pool of damage-associated molecular patterns (DAMPs), such as Hsp70 in the tumor microenvironment, thus prime adaptive antitumor immunity (52).

#### Thermogenesis

Experimental studies have shown that thermal damage occurs in two different stages. The first stage can lead to direct thermal damage of the tumor, which depends on the total energy of ablation, tumor components and microenvironment. Tumor cells are more vulnerable to heat damage as their specific biological characteristics and lower heat dissipation capacity and pH value (57). Another stage of damage is indirect damage, where residual heat leads to further tissue damage after the initial thermal stimulation has ceased (58).

#### Necrosis

Ablation is applied locally at extremely high or low temperatures, resulting in irreversible cell damage, ultimately leading to coagulative necrosis and apoptosis (59). Studies have shown that tumor cells cannot tolerate high temperatures well, and many tumor cells will undergo coagulative necrosis at 54-60°C (60). As described above, the electromagnetic microwave heats the material by stirring the water molecules, causing high-speed friction to generate heat. At temperatures above 60°C, proteins undergo rapid denaturation, which induces cell death through coagulation necrosis. Zhai et al. found that the ablation area of all MWA specimens showed features of nuclear shrinkage and cytoplasmic flow, which are typical characteristics of coagulative necrosis (61).

#### Apoptosis

It has been shown that apoptosis can be increased depending on temperature (62). When the temperature rises from 40°C to 45°C, important enzymes are inactivated and apoptosis may be triggered. In several studies of MWA, researchers found that apoptosis was significantly induced. In a liver microwave ablation experiment, it was found that caspase-3, a key enzyme of apoptosis, was significantly increased after MWA (63). And in microwave ablation therapy for patients with early tumor progression, researchers found that apoptosis-related miR-34a was significantly increased (64). All evidence indicated that MWA can induce apoptosis of cancer cells.

#### Pyroptosis

Pyroptosis is a pro-inflammatory programmed cell death caused by some inflammasomes, leading to the cleavage of gasdermin-D (GSDMD) and activation of interleukin-18 (IL-18) and interleukin-1beta (IL-1 β) (4, 65). Previous studies have shown that the expressions of caspase-1, GSDMD, IL-18, and IL-1β related to pyroptosis are up-regulated after heat treatment (radiofrequency ablation) of hepatic hemangioma, and induce pyroptosis of endothelial cells (66, 67). At present, there is no reports on the pyroptosis of cancer cells induced by MWA.

## Microwave ablation induced the release of ferroptosis-related molecular from tumor cells

**Figure 2:**
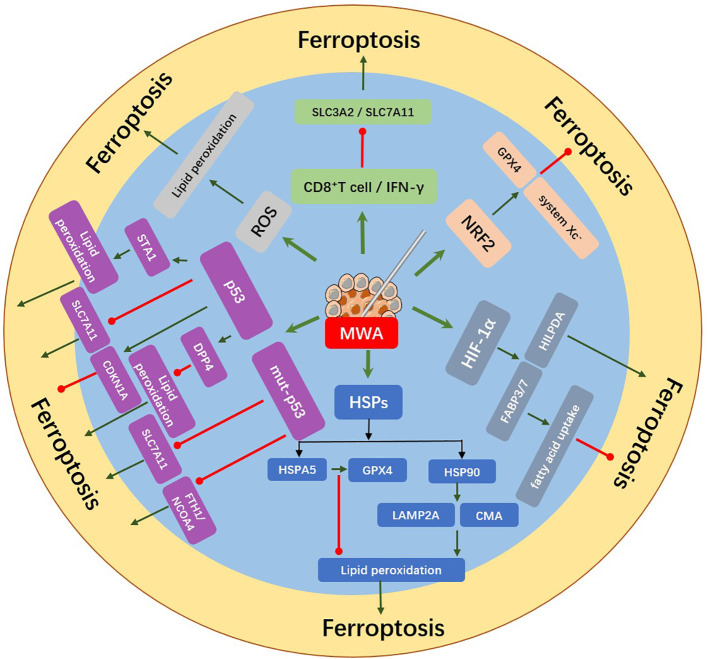
MWA induced the release of ferroptosis-related molecules from tumor cells. Upregulation of ROS, HIF-1α, HSPs, p53, NRF2, CD8^+^ T cell, and IFN-γ after MWA might regulate the occurrence of ferroptosis. MWA can induce lipid peroxidation by producing excessive ROS, which may further induce ferroptosis. p53 can induce ferroptosis by inhibiting SLC7A11 or promoting the activation of SAT1 and it can also inhibit ferroptosis by combining DPP4 or inducing the expression of CDKN1A. Mut-p53 can induce the sensitivity of ferroptosis by inhibiting the expression of SLC7A11 and FTH1/NCOA4. MWA upregulates HSPs expression. HSP90 can promote ferroptosis by regulating the stability of LAMP2A and CMA, while HSPA5 can inhibit ferroptosis by protecting GPX4 degradation. HIF-1α increases the expression of FABP3/7 and inhibits ferroptosis *via* promoting fatty acid uptake. The expression of HILPDA can promote ferroptosis. NRF2 can inhibit ferroptosis by promoting system Xc^-^ and GPX4. IFN-γ secreted by CD8^+^ T cells reduces SLC3A2 and SLC7A11, thus promoting lipid peroxidation and ferroptosis.

The process of ferroptosis in tumors and the biological response induced by microwave ablation shared the same molecules, such as ROS, protein 53 (p53), heat shock protein (HSP), hypoxia-inducible factor (HIF), and nuclear factor erythroid 2-related factor 2 (NRF2), therefore, we consider that microwave ablation may regulate ferroptosis of tumor cells. In this section, we explored the potential mechanism by which MWA might induce ferroptosis of cancer cells (**Figure 2**).

### MWA might regulate the ferroptosis of cancer cells by induced ROS

ROS are produced by normal physiological processes and plays a critical role in the signal of cell and tissue homeostasis (23). Sufficient studies found that the occurrence of ferroptosis is mainly due to the accumulation of iron and the consumption of antioxidant glutathione which leads to the increase of ROS, lipid peroxidation, and even cell death (68). Excessive iron will lead to the accumulation of ROS in cells (33). Iron chelating agents, such as Defeoxamine, can inhibit ferroptosis by inhibiting the overexpression of iron (24). In the procedure of iron metabolism, Fe^2+^ mainly mediates the production of ROS through the Haber-Weiss reaction and Fenton reaction, thus inducing the occurrence of ferroptosis (23). Ferroptosis could be caused by ROS-induced lipid peroxidation in cancer cells (3), therefore, MWA may induce ferroptosis of cancer cells by up-regulating ROS. Indeed, MWA can also induce lipid peroxidation by producing excess ROS. Specifically, ROS can extract electrons from PUFA to form PUFA radicals (PUFA•). These free radicals are in an unstable state and can rapidly interact with oxygen molecules to generate lipid peroxide radicals (PUFA-OO•), which are then extracted from other molecules by the Fenton reaction, and finally form lipid hydrogen peroxide (PUFA-OOH) (69). Therefore, MWA may lead to lipid peroxidation through ROS accumulation and the Fenton reaction, thereby regulating the ferroptosis of cancer cells.

### MWA might regulate the ferroptosis of cancer cells by induced p53

The p53 tumor suppressor gene is called the guardian of the genome because it can participate in the survival and division of cells (70). In addition to its effects on apoptosis, cell cycle, and autophagy, p53 can regulate ferroptosis by transcriptional or post-translational mechanisms (71, 72). Studies have found that heat stress can also induce the accumulation of p53 protein, thus MWA may regulate ferroptosis of cancer cells through p53. p53 regulates ferroptosis in some ways which can sensitize cells to ferroptosis by inhibiting the transcription of SLC7A11 (73). Furthermore, it has been shown that p53 can induce ferroptosis by inducing lipid peroxidation through transcriptional activation of spermidine/spermine N1-acetyltransferase 1 (SAT1) (74). Moreover, p53 can also inhibit NADPH oxidases (NOX)-mediated lipid peroxidation in colon cancer by directly binding to the dipeptidyl peptidase Dipeptidyl peptidase-4 (DPP4) or limit ferroptosis by inducing cyclin-dependent kinase inhibitor 1A (CDKN1A) expression in fibrosarcoma cells (71). Mutant p53 (mut-p53) occurs in a large proportion of cancers. Similar to wild-type p53 (wt-p53), mut-p53 can also sensitize cancer cells to ferroptosis, and research shows that different TP53 mutation types can induce increased sensitivity to ferroptosis (75). In terms of mechanism, studies have shown that mut-p53 makes cancer cells sensitive to ferroptosis by reducing the expression of SLC7A11 (72, 76). It has also been found that mut-p53 may make PDAC cells prone to ferroptosis by reducing the expression of ferritin heavy chain 1 (FTH1)/nuclear receptor coactivator 4 (NCOA4) (77). Eprenetapopt (APR-246) is a compound to shift mutant p53 and induces ferroptosis in DLBCL cells carrying wt-p53 and other forms of TP53 mutations (78). Meanwhile, it has been found that heat stress can also induce the accumulation of p53 protein. Han et al. found that heat stress significantly increased p53 protein levels in liver cells and hepatocellular carcinoma cells. In terms of mechanism, heat stress increased the half-life of p53 protein but not the expression of p53 mRNA or the activity of the p53 promoter (79). Although no direct evidence has been found, we consider that thermal stimulation induced by microwave ablation may adjust the ferroptosis of tumor cells by upregulation of p53 and MWA may also enhanced ferroptosis in mut-p53 tumors as ferroptosis of tumor cells could be influenced by p53 mutation types.

### MWA might regulate the ferroptosis of cancer cells by induced HSP

HSP include a large group of proteins such as HSP40, HSP70, HSP90, and other small families, which exhibit multiple functions in the tumor. HSPs are expressed and secreted into extracellular space by tumor cells (80). Research shows that HSP plays a crucial part in the genesis and development of ferroptosis, thus MWA might regulate the ferroptosis of cancer cells *via* HSP. Wu et al. found that HSP90 inhibitors reduced erastin/glutamate-induced ferroptosis in HT-22 cells, providing evidence that HSP90 is involved in mediating ferroptosis (81). Mechanistically, HSP90 promotes ferroptosis by regulating the stability of lysosomal-associated membrane protein 2a (LAMP2A) and chaperone-mediated autophagy (CMA) receptors (81). In addition, heat-shocked 70 kDa protein 5 (HSPA5) was found to negatively regulate ferroptosis in PDAC cells (82). Mechanistically, HSPA5 binds GPX4 and protects the degradation and lipid peroxidation of GPX4 protein (82). These studies support the idea that the induction of HSPs is related to the regulation of ferroptosis. MWA induced upregulation of HSP in cancer cells. HSP70 is known to protect the cell from heat exposure. In animal liver and tumor models, HSP70 is the main HSP in the ablative periphery after local thermal ablation. In addition, some studies have shown that MWA up-regulates the expression of HSP90. Zhai et al. have found that HSP90 overexpression can be found in peripheral tissues of liver carcinoma 24 h after MWA (61). Chen et al. found that HSP90 in the VX2 cells was significantly upregulated by MWA, and MWA combined with transforming growth factor-beta 1 (TGF−β1) and HSP90 inhibitors demonstrated the synergistic tumor treatment effect (83). Thermal ablation can enhance the expression of heat shock protein and change the antigenicity of the tumor. As mentioned above, HSPs were highly expressed in the sub-ablation site after microwave ablation. There is no direct evidence that MWA induced the occurrence and development of ferroptosis of cancer cells, however, MWA might regulate ferroptosis of cancer cells *via* altered HSP expression in the sub-ablation site.

### MWA might regulate the ferroptosis of cancer cells by HIF

Due to the rapid growth of tumor cells without an adequate blood supply, the tumor environment is characterized by hypoxia (84). The main regulator of hypoxia, HIF, is a spiral transcription factor that regulates genes involved in angiogenesis, glycolysis metabolism, and other biological process that are involved in cancer development and tumor growth (85). Microwave ablation may regulate the ferroptosis of cancer cells through a HIF-mediated pathway. HIF appears to play a crucial part in the regulation of ferroptosis. Hypoxia-induced upregulation of HIF-1α increases fatty acid binding proteins 3 (FABP3) and 7 (FABP7) in HT-1080 fibrosarcoma cells, thereby inhibiting ferroptosis *via* promoting fatty acid uptake to avoid subsequent lipid peroxidation. Endothelial Per-Arnt-Sim (PAS) domain protein 1 (EPAS1) activation promotes ferroptosis by upregulating hypoxia inducible lipid droplet associated (HILPDA) expression in renal cell cancer (RCC)-derived cells, thereby increasing PUFA generation and lipid peroxidation (7). Therefore, the valid control of HIF-mediated signaling is indispensable to keep lipid homeostasis. Restrain of the HIF-1α/SLC7A11 pathway is necessary for sorafenib-induced ferroptosis, whilst increased HIF-1α inhibits ferroptosis in hepatic stellate cells (HSC) (86). A lot of studies found that HIF is overexpressed in the tumor surrounding area after MWA. Duan et al. found that high levels of HIF-1α were also expressed at the ablation margins of MWA-induced livers, which may depend on secondary effects of hyperthermia (e.g., hypoxia due to endothelial injury or vascular thrombosis) (87). Wan et al. further found that high expression of HSP70 and HIF-1α could be found in the residual tumor cells around the ablation site (88). Li et al. also found that MWA can promote the expression of HIF-1α, thereby promoting angiogenesis by promoting the overexpression of vascular endothelial growth factor (VEGF), and accelerating the progression of tumor tissue invasion (89). In terms of mechanism, Chen et al. demonstrated that sublethal heat stress inhibited HIF-1α degradation by inducing O-linked-N-acetylglucosaminylation (O-GlcNAcylation) to upregulate HIF-1α expression in hepatocellular carcinoma (HCC) cells (90). In terms of existing studies, HIF has various effects on the ferroptosis of cancers, thus it is necessary to determine the role of HIF after MWA in the occurrence of ferroptosis of cancer cells in the future.

### MWA might regulate the ferroptosis of cancer cells by NRF2

NRF2 is a transcriptional factor and the function of NRF2 is to activate downstream antioxidant factors (91). It has been found that NRF2 plays a significant role in ferroptosis, thus MWA might regulate ferroptosis *via* NRF2 in cancer cells. Previous studies have shown that heat shock (40-42°C), similar to the temperature in the transition zone of microwave ablation, can induce increases in peroxiredoxin 3 (Prx3), GSH, and glucose-6-phosphate dehydrogenase (G6PD) levels, and largely depending on the antioxidant transcription factor NRF2 (92). A study reported that NRF2 silencing inhibited proteasome expression and activity, which is helpful to improve thermotolerance (93). They proposed that NRF2 inhibitors prevent heat resistance involving antioxidant and proteasome systems, and in combination with hyperthermia and NRF2 inhibitor induce tumor cells more sensitive to chemotherapy. Studies have shown that the expression of NRF2 is directly correlated to ferroptosis. Decreased expression of NRF2 can promote sensitivity to ferroptosis inducers (94, 95). These studies suggest that NRF2 inhibitors may be a viable target for inducing ferroptosis. Therefore, we concluded that microwave ablation of sublethal regions may affect the expression of ferroptosis through the activation of NRF2. At the same time, according to research findings, NRF2 inhibitors can be combined with hyperthermia, such as microwave ablation, to play the sensitization role of chemotherapy drugs, and enhance the anti-tumor effect by inducing the occurrence of ferroptosis, which provides new ideas for subsequent cancer treatment.

### MWA might regulate the ferroptosis of cancer cells by immune activation

Notably, recent research has found that ferroptosis is a fresh intersection between immunotherapy and MWA. Immune activation is an important component of MWA-mediated tumor killing, and CD8^+^ T cells can enhance ferroptosis in the tumor. The peripheral region of MWA exhibits immune infiltrates, including DCs, macrophages, NKs, neutrophils, and CD4^+^ and CD8^+^ T lymphocytes. Local thermal stimulation significantly increased the expression of costimulatory molecules, such as CD8, CD86 (B7-2), MHC Class II (MHC-II), and intercellular adhesion molecule-I (ICAM-I), which are involved in T cell proliferation and activation (96). Duan et al. demonstrated that MWA treatment alone increased the number of CD8^+^ cytotoxic T lymphocytes (CTLs) infiltrated within tumors compared to untreated tumors (97). Hou et al. demonstrated that MWA alone significantly increased the immune response to residual tumor proliferation and metastasis compared to the untreated group, including increased infiltration of effector T cells (CD45^+^ CD3^+^ CD8^+^) and increased secretion of anti-tumor cytokines interferon-gamma (IFN-γ) and tumor necrosis factor alpha (TNF-α) (98). Xu et al. demonstrated that MWA can observably increase in interleukin-2 (IL-2) and IFN-γ levels in patients who suffer from non-small cell lung cancer (NSCLC) 1 month after MWA (99). Here are some studies on the relationship between immunotherapy and ferroptosis. Some studies have found that CD8^+^ T cells activated by immunotherapy can induce ferroptosis by enhancing lipid peroxidation, and the induction of ferroptosis in cancer cells can contribute to the anti-tumor effect of immunotherapy (100, 101). IFN-γ secreted by CD8^+^ T cells reduces SLC3A2 and SLC7A11, two subunits of XC-, and affected cysteine intake by tumor cells, thus promoting lipid peroxidation and ferroptosis (100). SLC7A11 expression was found to be a negative correlation with the expression of CD8^+^ T cells and IFN-γ in tumors, and down-regulation of SLC7A11 can improve the prognosis of cancer patients (102). Given that MWA may be able to induce the increase of CD8^+^T cells and IFN-γ, we reasonably speculate that MWA can promote lipid peroxidation and iron prolapse of tumor cells. In addition, for tumors displaying anti-ferroptosis characteristics, the combination of ferroptosis inducers with immunotherapy and MWA may enhance tumor ferroptosis and increase the sensitivity to immunotherapy and MWA.

## Conclusion and perspectives

Recently, we gradually understand the principle, regulatory mechanism, and application of ferroptosis in cancer treatment, nevertheless, whether MWA regulates the ferroptosis of tumor cells is still unknown. By reviewing some recent studies, we found that the process of ferroptosis in tumors and the biological response induced by MWA shared the same molecules, therefore, we consider that MWA may regulate ferroptosis of the cancer cell. In this section, we explored the potential mechanism by which MWA might induce ferroptosis of cancer cells, indicating that ferroptosis exhibits one of the candidate mechanisms of MWA-medicated tumor killing. Several studies have shown that induction of ferroptosis is a new and useful therapy method in cancer treatment, therefore MWA combined with FINs may be a new therapeutic strategy in tumor treatments. In conclusion, further clarifying the correlation between MWA and ferroptosis and better understanding the mechanism of ferroptosis induced by MWA will contribute to the enhancement of the anti-tumor immune response of MWA.

## Author contributions

LY was responsible for collecting relevant research information and writing the review, while MC, JL and JMX were responsible for drawing the pictures. XY and ZGW are responsible for the revision of the paper. In addition, we have two corresponding authors in this article. QX and JL contributed to the study design and manuscript revisions. All the authors contributed to the article and approved the submitted version.
